# Chimeric Antigen Receptor Cell Therapy: Current Status and Its Potential in Aging and Alzheimer’s Disease

**DOI:** 10.3390/ijms26189009

**Published:** 2025-09-16

**Authors:** Maria Carolina Jurcau, Carina Diana Iovanovici, Anamaria Jurcau, Marius Militaru, Radu Bogdan Udrea, Alexandra Comanescu, Vharoon Sharma Nunkoo

**Affiliations:** 1 Faculty of Medicine and Pharmacy, University of Oradea, 1 Decembrie Square nr. 10, 410073 Oradea, Romania; 2 Doctoral School of Biomedical Sciences, University of Oradea, Universitatii Street nr. 1, 410087 Oradea, Romania; 3 Department of Psycho-Neurosciences and Rehabilitation, Faculty of Medicine and Pharmacy, University of Oradea, 1 Decembrie Square nr. 10, 410073 Oradea, Romania; 4 Department of Neuroscience, “Victor Babeș” University of Medicine and Pharmacy, Eftimie Murgu Square nr. 2, 300041 Timisoara, Romania

**Keywords:** immunosenescence, aging, Alzheimer’s disease, neuroinflammation, lymphocytes, macrophages, NK cells, CAR constructs, ICANS, CRS

## Abstract

With an aging population, there is a worldwide increase in the prevalence of neurodegenerative diseases. Alzheimer’s disease (AD) is the most prevalent form of dementia. Research focusing on aging has revealed a time-related accumulation of senescent cells that escape the cell cycle but remain metabolically active and spread the senescent traits to neighboring cells via the senescence-associated secretory phenotype. The accumulated senescent cells in various tissues are involved in the pathogenesis of several age-related conditions. As such, eliminating them would be an appealing anti-aging strategy. Following the high success rates of engineered chimeric antigen receptor (CAR)-T cells in hematological malignancies, the scientific community has tried to adapt the strategy to fight aging and age-related diseases. Research in this area is only in its infancy, but the results obtained from in vitro and animal models are encouraging. Due to the serious side effects of CAR-T cell therapies (cytokine release syndrome, immune cell-associated neurological syndrome) and because in AD the elimination of neurons with neurofibrillary tangles and amyloid aggregates should be avoided (given the limited regenerative potential of these cells), CAR macrophages, CAR regulatory T cells, or exosomes derived from these cells are a more promising approach.

## 1. Introduction

Improvements in medical and personal care have helped humans to live longer. The World Health Organization (WHO) estimates that 1.4 billion people will be older than 60 by 2030, and that this figure will double by 2050 [1]. Aging is an irreversible natural process associated in most cases with an increase in the prevalence of age-related diseases such as neurodegenerative diseases and musculoskeletal, metabolic, and cardiovascular disorders [2], which exponentially raise personal and hospital costs.

The increasing number of elderly people living worldwide has sparked increasing interest in research on aging mechanisms and anti-aging strategies [3]. As such, 12 hallmarks of aging have been identified, which can be grouped into primary hallmarks (genomic instability, telomere attrition, epigenetic alterations, loss of proteostasis, disabled macroautophagy), antagonistic hallmarks, reflecting a response to damage, such as deregulated nutrient sensing, mitochondrial dysfunction and cellular senescence, and integrative hallmarks, arising when the accumulated damage caused by the primary and antagonistic hallmarks can no longer be compensated and result in stem cell exhaustion, alterations in intercellular communication, chronic inflammation, and dysbiosis [4]. Although therapeutic interventions on each of these hallmarks affect other hallmarks as well, we do not yet have well-established treatment strategies to halt or even reverse aging [5].

Aging is the most prominent risk factor for neurodegenerative diseases, including dementia. Given the profound changes in the personality and intellect of a person with dementia, older adults fear developing this disease more than they fear cancer or even death [6,7]. There is an alarming growth in the prevalence of dementia, mainly in developing countries. About 13.8 million people are expected to live with dementia in the USA and 150 million people worldwide [8] by 2050. The predominant type of dementia, accounting for approximately two-thirds of cases, is Alzheimer’s disease (AD) [9].

Research focused on identifying interventions and drugs for treating AD has been marked by many trial failures and only approximately 1% success rate [10]. One of the reasons for this issue is an incomplete understanding of the pathogenesis of AD. Many of the proposed mechanisms overlap with the biology of aging hallmarks. The amyloid hypothesis can be regarded as a failure of proteostasis, the cholinergic hypothesis aligns with deficits in intercellular communication, the mitochondrial cascade hypothesis belongs to an antagonistic hallmark, and the neuroinflammatory hypothesis falls into the category of integrative hallmarks [11]. Starting in 1964 [12], the immunologic theory of aging has been widely discussed, and immunosenescence has been identified as harmful, leading to chronic, sterile, low-level inflammation—known as inflammaging [13]—which is associated with age-related conditions [14].

Neuroinflammation has been increasingly implicated in the pathogenesis of aging as well as neurodegenerative disorders, including AD [15]. Some scientists even recommend the expansion of the AT(N) biomarker framework (“A” for amyloid beta deposition in the brain, “T” for tau pathology, and “N” for neurodegeneration) to include neuroinflammatory (“I”) biomarkers [16].

Given the high success rates of chimeric antigen receptor (CAR)-T cells in inducing long-lasting remissions in oncology, mainly in hematological cancers, the idea of engineering immune cells to combat senescent cells or to eliminate abnormal protein depositions in neurodegenerative diseases is very appealing. The first animal experiments using engineered immune cells successfully cleared amyloid depositions and improved cognition [17,18].

The present paper aims to review the contribution of inflammatory pathways to aging and AD and to highlight the promise and challenges of cell-based therapeutic strategies as anti-aging approaches and disease-modifying treatments for Alzheimer’s disease.

## 2. Inflammation in Aging

### 2.1. The Development of Senescent Immune Cells

The immune system can be regarded as a firewall that protects the human body against foreign pathogens and eliminates damaged or abnormal cells, such as senescent or tumor cells [3]. However, immunity declines with age, leading to a decreased ability to respond to new pathogens [19], vaccines [20], and a loss of immunity to previously encountered pathogens such as the varicella-zoster virus [21]. The characteristics of aging of the innate immune system have been less comprehensively addressed, but extensive research on the senescence of the adaptive immune response has shown that T cells in particular undergo remarkable changes [3].

T cells account for about 70% of lymphocytes and 7–24% of immune cells in the blood [22]. Like all white blood cells, T cells are generated in the bone marrow and mature in the thymus, where they acquire special markers that determine their fate. T cells acquiring the cluster of differentiation (CD) 4 protein marker become helper T cells, while acquiring the CD8 marker turns them into cytotoxic (killer) T cells [2]. Some self-reactive T cells express forkhead box P3 (FoxP3) and develop into regulatory T cells (Tregs) that regulate self-tolerance and limit excessive inflammatory responses [23] through the production of anti-inflammatory cytokines such as interleukin (IL)-2, IL-10, IL-35, or transforming growth factor β (TGF-β) [24].

Naïve CD4^+^ T cells (CD4^+^TN) interact in the secondary lymphoid organs with antigen-loaded dendritic cells for activation, the specificity of the response being controlled by the peptides presented on major histocompatibility complex (MHC) class II molecules on the dendritic cells. Upon activation, naïve T cells differentiate into several stages of effector and memory cells by expressing co-stimulatory receptors, while their proliferation is driven by cytokines such as IL-7 for CD4^+^ cells and IL-15 for CD8^+^ T cells [25,26]. Different subsets of CD4^+^ T cells develop, such as T helper type 1 (Th1), T helper type 2 (Th2), T helper type 17 (Th17), and regulatory T cells (Tregs). These subtypes produce specific cytokines that elicit distinct immune responses. Notably, Tregs inhibit immune responses and thereby can prevent autoimmune diseases [27].

T memory cells transiently survey secondary lymphoid and non-lymphoid tissues. A subset of these cells develops into tissue-resident memory T cells and remain for the long term in non-lymphoid tissues [28]. Their tissue retention is due to the expression of a series of surface proteins. CD69 sequesters sphingosine 1-phosphate (S1P) receptor-1 from the surface of tissue-resident memory T cells and prevents their return into the S1P-enriched lymphatic system or blood [29]. CD49a and CD103 promote the adhesion of these T cells to collagen IV and E-cadherin, retaining these T cell populations in the tissues [30,31].

**Figure 1:**
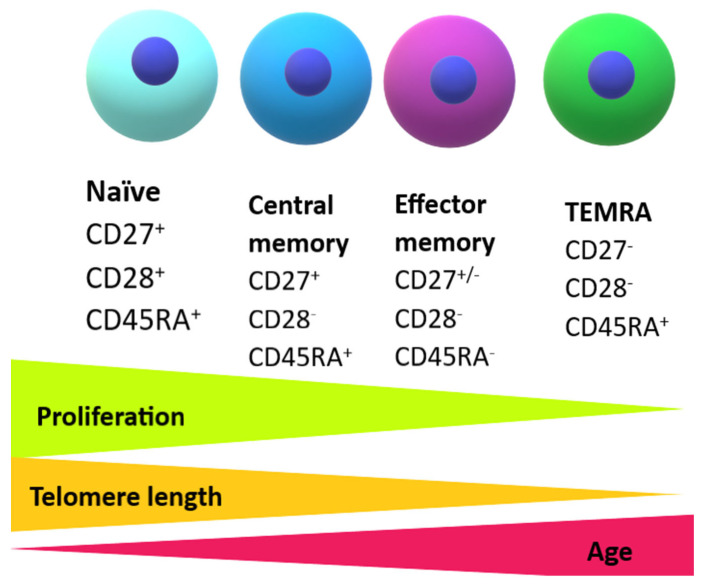
Model of T-cell differentiation during aging. Naïve T cells differentiate into memory and effector cells by losing the expression of the costimulatory receptors CD28, CD27, and CD45RA. However, when they reach an end stage, they regain expression of CD45RA and become terminally differentiated effector memory cells (TEMRA) with limited replicative capacity and shortened telomeres, and acquire NK-related functions that are highly cytotoxic [32].

A combination of technologies identifying telomeres and cell surface markers in subsets of T cells by multi-parameter flow cytometry enabled the identification of the pathways for differentiation of human T cells [32]. Both CD4^+^ and CD8^+^ naïve T cells express the co-stimulatory receptors CD27 and CD28 and have long telomeres. However, during differentiation into effector and memory cells, they alter the expression of their cell surface markers. Further, effector memory (EM) CD27^−^CD28^−^ T cells re-express cell surface CD45RA and develop into the EMRA T-cell subset (TEMRA—T effector memory re-expressing RA cells) [33] (Figure 1). CD27^−^CD28^−^ T cells express cell surface markers of T-cell senescence such as CD57 and KLRG1 (killer cell lectin-like receptor subfamily g), as well as intracellular molecules associated with cell cycle arrest and senescence (p16 and p21) [34].

The decline of the immune function can be attributed to age-related defects in the hematopoietic stem cells as well as to the involution of the thymus, which leads to a reduction of CD4^+^TN due to disturbances in T-cell receptor (TCR) gene rearrangement and a decrease in MHC molecules [35]. In the quiescent state, CD4^+^TN cells have low metabolic, transcriptional, and translational activities. Tribbles homolog 2 (TRIB2) regulates the high expression of protein kinase B (Akt) [36] and other transcription factors such as T-helper-inducing POZ/Krueppel-like factor (ThPOK); recombinant Runt-related transcription factor 2 (RUNX2) is also highly expressed. With age, ThPOK and TRIB2 expression decreases, resulting in increased IL-7-induced Akt phosphorylation and phosphatidylinositol-3-kinase (PI3K) hyperactivation [37]. Together with a reduction in HELIOS, a transcriptional repressor of T lymphocytes, these changes promote the differentiation of T cells into tissue-aggressive cells and enhance the phosphorylation of the signal transducer and activator of transcription 5 (STAT5), which results in an inflammatory microenvironment [38].

Metabolic dysfunction, with reduced biosynthesis of thymidine and purine, also negatively impacts the survival and proliferation of CD4^+^TN cells [39], as does mitochondrial dysfunction caused by the deficiency of mitochondrial transcription factor protein A (TFAM) and switching of the energy metabolism mode to glycolysis [40].

For yet incompletely understood reasons, naïve CD8^+^ T cells exhibit greater vulnerability to the effects of aging [41]. Alterations in the naive CD8^+^ T-cell pool are due to cell-extrinsic and cell-intrinsic changes that accompany the aging process. Age-associated mitochondrial dysfunction and ROS accumulation shortens the telomeres [42]. Epigenetic modifications also contribute to the remodeling of the CD8^+^ TN cell compartment. The activity of a series of transcription factors (NF-κB, STAT, TCF1, NRF1) is reduced with age in these cells [43]. NRF1 (nuclear respiratory factor 1), together with its transcriptional coactivator PGC-1α (peroxisome proliferator-activated receptor gamma coactivator 1-alpha), promotes transcription of genes involved in the electron transport chain and regulates TFAM1 [39]. Since CD8^+^TN rely mainly on oxidative phosphorylation for homeostatic expansion, these changes likely contribute to their defective proliferation and may also induce epigenetic modifications [44].

Nonetheless, T-cell numbers are maintained throughout life in the absence of new input of thymus-derived naïve cells due to the IL-7- and IL-15-induced homeostatic proliferation of T cells and due to the repeated rechallenge by antigens [26,45]. Unfortunately, the continuous proliferation of T cells leads to telomere shortening and disrupts the ability to upregulate the enzyme telomerase, causing the loss of their proliferative potential (replicative senescence) [46]. Recently, a new mechanism for telomere maintenance independent of telomerase activity has been described, namely the transfer of telomeres together with DNA recombinant factor Rad51 via extracellular vesicles from antigen-presenting cells. This approach led to the elongation of recipient T-cell telomeres by about 3000 base pairs, but occurred only in naïve and central memory T cells, and to a much lesser extent in effector T cells [47].

### 2.2. Altered Function of Senescent T Cells

Senescent cells within both CD4^+^ and CD8^+^ compartments have low proliferation rates, but high secretory activity, expressing high levels of interferon (IFN)-γ, TNF-α, other pro-inflammatory cytokines, as well as cytotoxic proteins such as granzyme and perforin [48]. A series of stress proteins called sestrins, coded by the *Sesn1*, *Sesn2*, and *Sesn3* genes, are expressed and activate mitogen-activated protein kinase (MAPK) in a sestrin-activated MAPK activation complex (sMAC), which activates independently of mTOR (mammalian target of rapamycin) activity [49] and blocks antigen-specific proliferation of senescent CD4^+^T cells [50].

The senescent CD8^+^ cell population also express high levels of sestrins [51], express various activating and inhibitory NK receptors and their adaptor molecules [52], and lose TCR signaling-related molecules, leading to a reduced TCR signaling-induced proliferative capacity.

### 2.3. Immune Surveillance of Senescent Cells

A common feature of senescent cells in the body is the secretion of a series of cytokines, chemokines, matrix metalloproteinases, and growth factors, collectively known as the senescence-associated secretory phenotype (SASP) [5]. Of the cytokines, the role of the anti-inflammatory ones such as TGF-β is largely unknown [53], but the pro-inflammatory ones create an inflammatory microenvironment that can stimulate other cells, for example tumor cells, to grow and proliferate. At the same time, they enhance tumor immunity by recruiting immunocompetent cells like cytotoxic T cells, natural killer cells, or macrophages; remodel the cell surface proteome; and activate IFN signaling and MHC-I antigen presentation [54]. The cells’ immunopeptidome altered by senescence, which facilitates recognition by immune cells, consists, among others, of the expression of higher levels of endogenous retroviral genes [55]. This is likely due to the reactivation of the expression of retrotransposable elements and endogenous retroviral genes by the global DNA hypomethylation status, a hallmark of senescence [4], with focal DNA hypermethylation at CpG (regions of DNA where a cytosine nucleotide is followed by a guanine nucleotide in the linear sequence of bases along its 5′ -> 3′ direction) island promoters [56].

The immune system is the major player in clearing senescent or damaged cells [57]. For example, senescent liver cells are removed prior to malignant transformation by CD4^+^ T cells and macrophages [58]. Faced with the host’s innate immune system, senescent cells increase the expression of CD47, which acts as a “don’t-eat-me” signal by binding to the inhibitory receptor signal-regulated protein α (SIRPα) on macrophages [59,60].

However, the gradual decline in the function of T cells with age and the increase in the number of CD8^+^ T cells expressing the inhibitory receptor NKG2A facilitate the escape of senescent cells from immune surveillance. Senescent human skin fibroblasts and endothelial cells express high levels of the MHC-Ib molecule HLA-E due to the activation of the p38MAPK pathway [61], and HLA-E suppresses the function of NKG2A-positive CD8^+^ T cells [62]. Another molecule highly expressed by senescent cells that facilitates their escape from elimination by the immune system is the immune checkpoint molecule PD-L1 [63], which leads to stronger inflammatory phenotypes and promotes aging phenotypes in neighboring cells [64].

## 3. Neuroinflammation in Alzheimer’s Disease

Alzheimer’s disease does not have a “root cause”, but is rather a multifactorial disease in which molecular, genetic, and environmental factors converge and lead to progressive neurodegeneration. The limited efficacy of anti-amyloid monoclonal antibodies in AD suggests that additional mechanisms related to abnormal tau processing [65], mitochondrial dysfunction [66], synaptic degeneration [67], and neuroinflammation [15] should be further explored to fully understand the pathogenesis of AD. Research increasingly highlights the intertwined neuroinflammatory signaling pathways involved in the onset and progression of AD. Of the ongoing phase II clinical trials, approximately 20% target these inflammatory mechanisms [68].

The CNS has several cells with immune functions. Microglia mainly drive the innate immune response, functioning as brain-resident macrophages that constantly survey the brain parenchyma and phagocytize cellular debris, pathogens, and damaged neurons [69]. When detecting damage signals, microglia switch to an activated state and release pro-inflammatory cytokines. In healthy brains, the process is terminated shortly after eradication of the inflammatory insult. If, however, microglial activation is dysregulated or prolonged, the excessive release of neuroinflammatory factors becomes neurotoxic and attracts peripheral T cells into the CNS [70]. Although not considered immune cells, astrocytes communicate with microglia and are able to mount an inflammatory response that leads, among other things, to a permeabilization of the blood–brain barrier (BBB) [71], thereby promoting the infiltration of the brain with peripheral immune cells.

With aging, circulating immune cells accumulate in the CNS and perpetuate a chronic inflammatory state [72]. Although the accumulation of Tregs could appear protective, it impairs recovery from inflammatory insults by suppressing IFNγ-producing cells and interfering with Tregs trafficking to the CNS [70]. Brain-resident Tregs express CD69 [73] and ST2 (the receptor for IL-33) [74], as well as the serotonin receptor Htr7, which is responsible for the expansion of this cellular population upon serotonin administration [75]. These resident Tregs produce neuropeptide Y, as well as amphiregulin and osteopontin, which are responsible for mediating the interaction between Tregs and microglia and for suppressing reactive gliosis [76].

### 3.1. Genome-Wide Association Studies Support the Contribution of Neuroinflammation in the Development of Alzheimer’s Disease

Many genome-wide association studies (GWASs) have identified a series of genetic variants linked to the susceptibility of an individual to AD beyond the well-known APOEε4 allele [77]. However, in Table 1, we show only those genetic susceptibility loci that are linked to neuroinflammation.

### 3.2. The Innate Immune System in Alzheimer’s Disease

The brain’s innate immune cell is the microglial cell that acts similarly to macrophages in the periphery. In the early stages of AD, microglia exhibit protective roles by phagocytosing Aβ aggregates [90], removing dysfunctional synapses, regulating neuronal connectivity [91], and providing trophic support to neurons [92].

However, with disease progression, microglia become chronically activated and switch to a pro-inflammatory phenotype that releases pro-inflammatory cytokines (TNF-α, IL-1β, IL-6) and reactive oxygen species [15], become senescent and release SASP factors while failing in their clearance of Aβ species [93], and, via the complement system, perform excessive synaptic pruning, leading to loss of functional synapses [94]. Through these actions, in later stages of AD, microglia promote tau phosphorylation and spread [95] and drive local inflammation [96].

### 3.3. The Adaptive Immune System in Alzheimer’s Disease

The pathogenic role of the brain’s resident immune cells, the microglia, is well established in AD [15,97]. However, microglia recognize only a limited array of antigens through germline-encoded receptors [98], while T and B cells recognize virtually unlimited molecular patterns through receptors generated by rearrangement of gene segments encoding antigen-binding sites [99]. Nonetheless, the relevance of adaptive immune responses in AD is just beginning to be elucidated [100,101].

B cells recognize antigens through rearranged B cell receptors (BCR), while T cells recognize antigens presented on major histocompatibility complex (MHC) molecules through heterodimeric T-cell receptors (TCRs). T cells include cytokine-producing CD4^+^ T helper cells, CD8^+^ cytotoxic T cells, and regulatory T cells (Tregs) [98]. After antigen recognition, T and B cells expand clonally with similar or identical BCR or TCR sequences. The contribution of the adaptive immune responses to the pathogenesis of AD is highlighted by the markedly reduced amyloid pathology in *Rag*^−/−^ mice, which, due to their incapacity for gene rearrangement, lack mature B and T cells [102].

#### 3.3.1. B Cell Responses Against Amyloid Beta and Tau

Studies investigating serum and CSF levels of anti-Aβ antibodies yielded conflicting results, although most of them showed increased levels of anti-Aβ antibodies [103]. However, similar antibodies are found in healthy individuals as well. In fact, aducanumab was cloned from the immunoglobulin of a healthy aged individual [104]. Moreover, healthy individuals also harbor antibodies that recognize phosphorylated tau [105].

Under normal conditions, B cells are present in low numbers in the CSF and brain parenchyma. They enter the CNS via the BBB, exiting postcapillary venules and entering the perivascular space from where they can further penetrate the brain parenchyma, through the CSF, by crossing the choroid plexus, or via extravasation from subpial venules. To reach the parenchyma from the CSF, B cells cross the glia limitans [98]. Recent studies have shown that in young adult mice, B cells are not blood-borne, but derive from B cell progenitors in the skull bone marrow, from where they migrate into the meninges through ossified vascular channels [106]. The meningeal B cells of aged mice are mainly blood-borne and have a characteristic phenotype, known as age-associated B cells [106]. Skull bone marrow-derived B cells exposed to CNS antigens develop into CNS-tolerant B cells, while age-associated B cells are more prone to react to CNS antigens by producing specific antibodies, such as anti-Aβ and anti-tau antibodies [107].

#### 3.3.2. T Cells in Alzheimer’s Disease

T cells infiltrating the brain of patients with AD were reported decades ago [108], occurring mainly in the entorhinal cortex and amygdala. These T cells originate from the periphery [109] and are mainly cytotoxic CD8^+^ T cells [110]. However, clonally expanded CD8^+^ T cells were also found in the CSF and peripheral blood of patients with AD. These cells were characterized as T effector memory cells re-expressing CD45RA^+^, also known as TEMRA [111].

The age-related enrichment of the brain in inflammatory cytokines driven by chronic, sterile, low-level inflammation (inflammaging) [5] promotes T-cell infiltration and residence [112]. The BBB dysfunction associated with increasing age promotes T-cell infiltration of the brain parenchyma [113]. The infiltrates occur mainly around Aβ plaques. In vitro studies have shown that Aβ-reactive Th1 cells produced IFNγ and activated microglial response to Aβ plaques [114], while human studies confirmed the expansion of a T helper cell population highly responsive to IFN signaling [115]. However, the effect of these helper cells is controversial: while some studies have found increased Aβ clearance in their presence [114], other studies have reported an increased burden of amyloid pathology [116]. Overall, it appears that infiltrating T cells augment neuroinflammation but have no significant effect on Aβ or tau aggregates. Antibody depletion of CD8^+^ cytotoxic T cells in aged APP-PS1 mice led to microglial expansion in the hippocampus but had no effect on amyloid pathology [110], while depletion of both CD4^+^ and CD8^+^ T cells in THY-Tau 22 mice decreased the magnitude of CNS inflammation and prevented behavioral deficits, but did not impact tau pathology [117].

T cells responsive to Aβ and tau were also found in the peripheral blood of patients with AD [118]. Several routes for priming of T cells have been identified:

- Brain antigens transported via the CSF can access the nasal mucosa via the cribriform plate, from where they are further drained by the lymphatic circulation into the deep cervical lymph nodes [119].
- Antigens in the CSF can also be drained directly by meningeal lymphatic vessels into the deep cervical lymph nodes [119].
- CNS antigens from the CSF can be captured by meningeal dendritic cells, which also reach the deep cervical lymph nodes through the meningeal lymphatics and contribute to priming of the immune cells [119].

In addition, the number and function of regulatory T cells, crucial in the homeostasis of the immune system and in preventing excessive immune responses, have been reported by most studies to be reduced in AD [73,120]. Tregs also exhibit different phenotypes compared to healthy individuals, with a reduced expression of the surface antigen CD25, which is important for Treg survival and expansion [121]. While microglial activation contributes to clearance of misfolded proteins and Aβ soluble and fibrillary forms, sustained activation causes microglia to skew towards a defective subtype that damages the CNS [69]. The suppressive function of Tregs regulates the inflammatory microglial phenotype, as shown by studies that increased the number and function of Tregs through ex vivo expansion and adoptive transfer [122,123].

## 4. CAR Cell Therapy

Given the significant involvement of inflammatory pathways in the pathogenesis of both aging and neurodegenerative diseases, including AD, a series of therapeutic strategies targeting inflammation have been investigated. Following the significant success of chimeric antigen receptor T (CAR-T) cell therapy in the field of malignant tumors, modulating immunity with adoptive cell therapy is an area of active research in neurodegenerative diseases and as an anti-aging strategy.

### 4.1. The CAR-T Cell Construct

In 1989, Gross and coworkers designed a chimeric T-cell receptor (TCR) consisting of the TCR constant domain fused with the antibody variable domain and transfected it into CD8^+^ T cells, which conferred antibody-like specificity to these T cells [124] and enabled them to be activated and execute their effector function when encountering the targeted antigen. Subsequently, the technique was developed and used to successfully treat hematological malignancies, leading to the FDA approval of tisagenlecleucel, the first chimeric antigen receptor (CAR)-T cell product, for treating patients with relapsed/refractory B-cell acute lymphoblastic leukemia [125]. To date, 6 CAR-T cell therapies have received FDA (Food and Drug Administration) and EMA (European Medicines Agency) approval for the treatment of hematological cancers [126].

**Figure 2:**
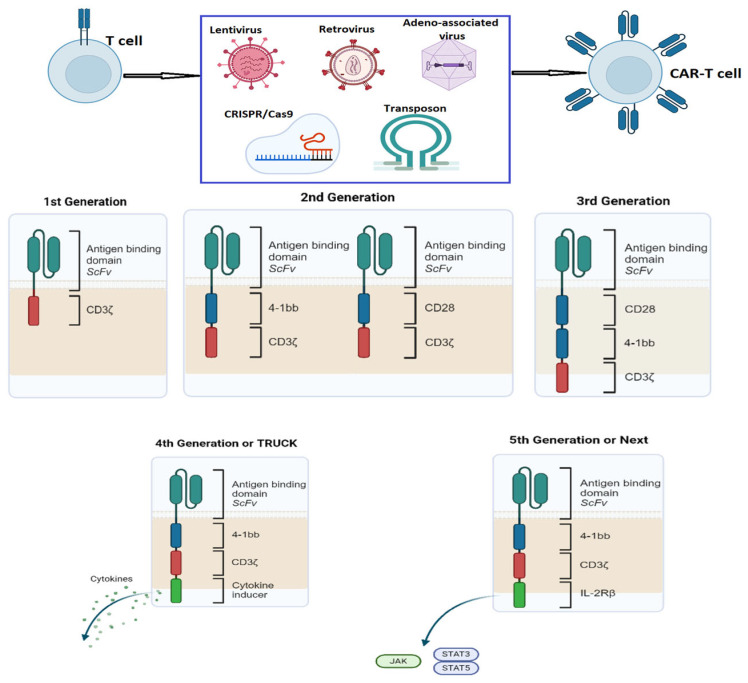
Generation of CAR-T cells. First-generation CARs contained only the CD3ζ domain that initiated the intracellular signaling but, due to the lack of a co-stimulatory signal, had limited persistence and ability to expand. Second-generation CARs contained CD3ζ and a co-stimulation signal such as CD28 or 4-1BB. As such, they had improved persistence, expansion, and cytotoxicity. Third-generation CARs had a second costimulatory domain (the first being CD28 or 4-1BB and the second being CD28, 4-1BB, or OXO40). They were superior in terms of T-cell expansion, persistence through cytokine production, and proliferation speed. Fourth-generation CARs, also called TRUCKs (T-cells redirected for universal cytokine-mediated killing), have a cytokine-induced domain that activates the transcription factor NFAT to produce cytokines after antigen recognition and thus modulate the immune effects. Fifth-generation CARs have an inactivated T-cell receptor alpha constant (TRAC) gene through gene editing, which leads to the removal of the TCR alpha and beta chains and the creation of a truncated cytoplasmic IL-2 receptor β-chain domain with a binding site for STAT3 transcription factor. Antigen activation triggers three synergistic signals through TCR CD3ζ, co-stimulatory CD28, and cytokine JAK–STAT3/5 signaling, which drive T-cell activation and proliferation. Created in BioRender. Jurcau, A. (2025) https://BioRender.com/u20ffdq.

Conventional CAR is a recombinant receptor composed of the antigen-binding domain (usually a single-chain variable fragment—scFv) cloned from a monoclonal antibody (mAb) fused to intracellular signaling domains via a spacer or hinge. The spacer is the flexible part of the CAR and is responsible for the flexibility of the receptor in the antigen recognition process and can modulate the downstream signaling events to some extent [127]. The intracellular domain consists of a signaling domain and co-stimulatory domains. Upon antigen engagement by the CAR, the intracellular signaling domain, most commonly CD3, triggers a transcriptional pathway dependent on the nuclear factor of the activated T cell (NFAT) to elicit cytotoxicity. The co-stimulatory molecules (CD28, CD27, 4-1BB, or OX40) influence the cytotoxicity rate and the development of the immunological memory phenotype [128,129] and ultimately determine the receptor’s activation efficiency (Figure 2).

To produce autologous CAR-T cells, the patient’s T cells are collected through leukapheresis after the patient has abstained from all medication for 2 weeks to confer the best viability to the harvested cells [127]. Following this step, the CAR gene is introduced into the T cells using a lentivirus or a retrovirus, although other techniques are under research, such as transposons, nanoparticles, zinc fingers, or CRISPR/Cas9 (clustered regularly interspaced short palindromic repeats/Caspase9) [130]. The engineered T cells are then expanded ex vivo using a cocktail of cytokines, like IL-2, IL-7, or IL-15 [131], while the patient undergoes lymphodepletion in preparation for receiving the adoptive cell transfer to enhance CAR-T cell expansion, persistence, and activity [132].

The continuous refinement and evolution of CAR design and generation led to enhanced specificity, efficacy, and safety of CAR-T cell therapies. Currently, five CAR generations are recognized in the field of immunotherapy (Figure 2):

1. The first generation of CAR-T cells had an extracellular scFv domain linked to an intracellular CD3 signaling domain. These T cells had a low survival rate and could not produce a long-lasting immunological response.
2. The second generation of CAR molecules was developed by adding a co-stimulatory domain (CD28 or 4-1BB) to the CD3 signaling domain, leading to increased resistance to exhaustion of the engineered cells [133].
3. Third-generation CAR constructs used 2 co-stimulatory domains, which increased efficacy, proliferation rate, and durability of the transferred cells [134].
4. The fourth generation is also known as TRUCK cells (T Redirected Universal Cytokine Killer Cells) and is engineered to also produce cytokines (IL-2, IL-15, IL-18), enzymes, and other biochemical substances that enhance the anti-tumor effect and the persistence of CAR-T cells in the tumor microenvironment [135].
5. Fifth-generation CARs include additional elements that enable engineered cells to recognize multiple targets even in an environment with low antigen density [136].

Other specific CAR designs and strategies also boost the specificity and efficacy of the transferred cells. Tandem CAR-T cells contain 2 bispecific ligand-binding domains and are activated if one or the other antigen-binding domain binds their antigen. Dual CAR-T cells contain 2 different CARs, and the immune response is activated only when both antigens are expressed on the target cell [127]. UCARs (universal CAR-T cells) use allogeneic T cells from healthy donors and significantly reduce the costs and time needed for manufacturing the immunotherapeutic cells, although the risks of developing graft-versus-host disease or host-versus-graft rejection are higher than for the classical approach. By incorporating an additional component that separates the antigen-targeting domain from the T-cell signaling unit, the engineered cell can theoretically target any antigen. One such component is the biotin-binding immune receptor (BBIR), which recognizes a variety of biotinylated antigens [137]. However, UCARs containing murine scFv may elicit a human anti-mouse IgG antibody (HAMA) response against the CAR construct, leading to anaphylactic shock or cardiac arrest, a risk that can be lowered by using humanized scFvs [127]. The split, universal, and programmable (SUPRA) CAR construct is composed of the universal receptor expressed on T cells (zipCAR) and the tumor-targeting scFv adaptor (zipFv). The zipCAR combines a leucine zipper serving as the extracellular domain with intracellular signaling domains, while the zipFv adaptor molecule is formed by fusing a scFv that binds to the tumor antigen with a complementary leucine zipper. As such, complementary leucine zippers activate the zipCAR on T cells [127,137]. Being modular, SUPRA CARs can target multiple antigens, and by employing a competitive zipFv able to bind to other zipFvs, the function of the zipCAR can be finely tuned and restrained when not required [138].

### 4.2. Current Applications of CAR-T Cell Therapy

CAR-T cell-based drugs are currently approved by the FDA and the European Medicines Agency (EMA) in hematological cancers. They are less effective against solid tumors due to the heterogeneity of the tumors and the immunosuppressive microenvironment [139]. Other 2 types of CAR-T cell-based drugs are in clinical trials but not yet approved in the USA or the EU. Equecabtagene autoleucel has an anti-BCMA CAR-T cell structure and was developed for patients with relapsing or refractory multiple myeloma who have progressed after ≥3 lines of treatment, including a proteasome inhibitor and an immunomodulatory agent [130]. Given the clinical efficacy in the FUMANBA trial (NCT05066646), it was approved for clinical use in China in 2023 [139]. Preliminary results also indicate a good response in relapsing or refractory aquaporin4-IgG seropositive neuromyelitis optica [140]. Actalycabtagene autoleucel is a novel humanized anti-CD19 CAR-T cell therapy evaluated in diffuse large B-cell lymphoma and acute lymphoblastic leukemia [141]. Following the good response rates of B-cell lymphomas and leukemias in the pooled analysis of the results from phase I and II clinical trials, it was approved in 2023 in India for clinical use [142].

### 4.3. Side Effects of CAR-T Cell Therapy

Aside from common limitations, such as loss of target antigen, decreased antigen identification, CAR-T cell exhaustion, and insufficient infiltration in solid tumors [143], of CAR-T cell therapies are also associated with serious side effects.

Cytokine release syndrome (CRS) develops in 30–100% of patients receiving CAR-T cell therapy [144]. Clinically, it manifests as hyperthermia with disturbed oxygenation and cardiovascular function. Its pathophysiology appears to rely on pyroptotic cell death induced by the release of granzyme B by CAR-T cells, which activates caspase-3 in the target cells followed by cleavage of gasdermin E and formation of pores in the target cell membrane [145] with release of IL-2Rα, IL-6, IL-8, IL-10, or IL-15 [146].

Immune cell-associated neurological syndrome (ICANS) is less common than CRS, affecting only half of patients undergoing CAR-T cell therapy for malignancies. It presents with frontal lobe dysfunction [147], language disorders and agraphia, seizures, or fulminant brain edema [148]. Its pathophysiology also relies on the production of a large variety of pro-inflammatory factors and cytokines by CAR-T cells, macrophages, or myeloid cells and weakening of the blood–brain barrier (BBB), leading to infiltration of the CNS with CAR-T cells and other immune cells and astrocytic and microglial activation [149].

Cognitive impairment is a common complication in CAR-T cell recipients, occurring mainly during the first week after treatment, and has been reported to improve [150] or worsen [151] over time.

### 4.4. Other CAR Cell Constructs

Other immune cells can also be genetically engineered to fight against cells expressing certain antigens. Chimeric antigen receptor macrophages (CAR-Ms) have been reported to phagocytose tumor cells [152] and are currently being evaluated in a phase I clinical trial (NCT04660929) for HER2-overexpressing solid tumors [153,154]. The extension of CAR-M technology to non-oncological conditions is still in its infancy, although very appealing. Regulatory T cells, expressing CD4, CD25, and FOXP3, are a subset of T cells acting as immunomodulators [133] and able to suppress immune responses via direct interaction with immune cells or by producing IL-10, TGF-β, or other immunosuppressive cytokines [155]. To date, CAR-Tregs are being developed for the treatment of graft-versus-host disease, diabetes mellitus type 1, rheumatoid arthritis, inflammatory bowel disease, asthma, vitiligo, hemophilia, and multiple sclerosis [133]. Moreover, generation of allogeneic CAR-Tregs by removing TCR could avoid the graft-versus-host reaction and could be used in patients in whom autologous cell therapy is impossible [156].

## 5. The Potential of CAR Cell Therapy Against Senescent Cells

Senescent cells exit the cell cycle and are resistant to apoptosis, but remain viable and metabolically active [4]. As such, they tend to accumulate in tissues and spread the senescent features to neighboring cells in a paracrine manner by secreting a series of chemokines, cytokines, and growth factors collectively referred to as the senescence-associated secretory phenotype (SASP) [5]. Through the various molecules of the SASP, senescent cells recruit immune cells such as macrophages, T cells, NK cells, or neutrophils [157].

Although neurons do not divide, they can acquire a senescence-like phenotype in the cortex and other important regions, a feature more expressed in AD [158]. Other cell types, such as astrocytes, oligodendrocytes, microglia, neural progenitor cells, and microglia, do exhibit senescence [159]. Studies performed in rodents, primates, and human tissues showed that senescent cells are not uniformly distributed across the CNS, but tend to accumulate in specific brain regions:

- The hippocampus [160]
- Cerebral cortex [161]
- In the microglia and oligodendrocytes of white matter [162]
- In the subventricular zone and dentate gyrus [163]

The percentage of senescent cells in the brain is difficult to estimate due to individual differences, the variable detection methods, and markers used by different researchers. However, Dehkordi et al. found an average of 4.2% senescent excitatory neurons in postmortem human tissues (varying between 0% and 20%) in the prefrontal cortex [164]. Other studies do not provide exact percentages, but emphasize the statistically significant difference in the number of senescent brain cells between young and elderly animals.

As for immune cells, distinct subsets of immune cells clear senescent cells developing as a result of DNA damage response, genomic instability, mitochondrial dysfunction, oxidative stress, inflammation, or inactivation of tumor suppressor genes [165]. Natural killer (NK) cells have various subpopulations able to recognize and eliminate malignant and virus-infected cells [166]. In addition, via their receptors, they can interact with ligands expressed on senescent cells and release granzyme and perforin [167]. The SASP initially enhances the NK cell-mediated immune response, but excessive accumulation of senescent cells and increased levels of SASP factors inhibit immunity [168]. Centenarians exhibit significantly increased activity of NK cells compared to middle-aged individuals [169]. The role of T cells in immune surveillance and healthy longevity is crucial. CD4^+^ T cells recognize MHC II molecules on senescent cells and directly eliminate them, while CD8^+^ T cells recognize the MHC I molecules on these cells [165]. Depleting pathogenic subsets and enhancing protective T cells can reset the immune system tolerance and can be a valuable rejuvenating approach [170]. Macrophages, as essential components of the host’s innate immune system, are recruited by senescent cells via SASP factors and initiate local inflammation [171]. However, with aging, some senescent cells can escape immune surveillance, as happens, for example, via upregulation of CD47, which exerts inhibitory action against macrophages [172]. A crucial connection between the innate and adaptive immune systems is provided by the invariant natural killer T cells (iNKTs), which enhance the removal of senescent cells through cytotoxin secretion and through regulation of the activity of other immune cells [173]. Although they account for only 0.001–1% of peripheral blood cells, they are able to recognize and be activated by exogenous and endogenous lipid antigens, particularly glycolipids [174], and secrete IFN-γ, TNF-α, or IL-4, through which they stimulate other immune cells to fight infections, autoimmune diseases, and tumorigenesis, or eliminate senescent cells [175].

In conclusion, eliminating or neutralizing these senescent cells could be a promising therapeutic strategy to delay aging and prolong a healthy lifespan [176]. The current anti-aging strategies can be categorized into 3 categories:

(a) Elimination of senescent cells (senolysis)
(b) Neutralization of the SASP (with senomorphics)
(c) Immune-based clearance of senescent cells

Senolytics target a series of signaling pathways of senescent cells and disrupt their anti-apoptotic mechanisms by using mainly small molecules that need repeated administration [177]. Senomorphics also target signaling pathways that lead to the production of the cytokines and chemokines of SASP, but healthy cells often use the same pathways. As such, the targeted cellular population is uncertain [178]. Immunotherapy could target senescence-specific surface antigens and successfully eliminate senescent cells with minimal off-target effects [179]. Antibodies against dipeptidyl peptidase 4 (DPP4) [180], antibodies conjugated with drugs directed against beta-2-microglobulin [181], or a vaccine against glycoprotein nonmetastatic melanoma protein B (GPNMB) [182] have been shown to effectively eliminate senescent cells expressing these antigens. Immune checkpoint blockade is increasingly used in cancer treatment and could be successful as an antiaging strategy as well. With age, there is a gradual accumulation of senescent cells expressing programmed cell death ligand 1 (PD-L1) [63]. The interaction between programmed cell death protein 1 (PD-1) and its ligand (PD-L1) modulates the response of the immune system to various aggressions. Nonetheless, the upregulation of PD-L1, as occurs in certain malignant cells, enables these cells to avoid being recognized and eliminated by the immune system [183]. Using PD-1/PD-L1 immune checkpoint blocking agents can eliminate senescent cells overexpressing PD-L1 [64]. This was proven in animal models of naturally aged mice and nonalcoholic steatohepatitis [64] and in patients with lung cancer treated with PD-1 inhibitors (nivolumab, pembrolizumab) or anti-PD-L1 inhibitors (atezolizumab), who experienced hair repigmentation [184].

### 5.1. CAR-T Cell Therapy Against Senescent Cells

The effectiveness of CAR-T cells as senolytics depends on accurately identifying surface antigens expressed only by senescent cells. This allows the precise design of a specific CAR construct. Most CARs target proteins and glycosaminoglycans located on the cell surface (Table 2), but more recently, it became possible to target the intracellular peptide p16 [185]. Senescent cells display p16-derived peptides in major histocompatibility complex (MHC)-peptide complexes on the cell surface. TCR-mimic antibodies that bind these peptide-MHC complexes can be fused into a CAR scaffold, the intracellular domain of which drives T-cell activation [185].

The urokinase plasminogen activator receptor (uPAR) is poorly expressed in vital tissues as opposed to uPAR -positive cells that are non-proliferative and express the SASP factor IL-6 [196]. As shown by Amor et al., anti-uPAR CAR-T cells are able to clear lung adenocarcinoma cells that enter a senescent state following combined CDK4/6 and MEK inhibition, as well as reverse liver fibrosis induced by both carbon tetrachloride and non-alcoholic steatohepatitis [188]. Moreover, CAR-T cells directed against uPAR-positive cells improved metabolic dysfunction and glucose tolerance in aged mice and mice fed a high-fat diet [197]. Other antigens highly expressed in senescent cells and not in normal cells are the natural killer group 2 member D ligands (NKG2DLs), which help senescent cells escape endogenous natural killer cell-mediated immune clearance [61]. CAR-T cells targeting NKG2DLs successfully eliminated senescent cells in aged mice and nonhuman primates [195] without serious side effects, a finding consistent with their good safety profile in clinical trials for cancer [198]. “Armored” CAR-T cells expressing additional proteins (cytokines or cytokine receptors) show enhanced effector function and prolonged persistence [199]. By adapting these technologies, engineered senolytic CAR-T cells could not just counteract but take advantage of SASP factors [200]. Nonetheless, further studies of potential side effects in an elderly population are still required before this strategy can be translated into clinical trials.

### 5.2. CAR-NK Cell Therapy Against Senescent Cells

CAR-NK cell therapy could offer advantages over CAR-T cell therapy such as enhanced safety and feasibility for “off-the-shelf” production [201]. The potential of NK cells has been evaluated in malignancies, infectious diseases, and autoimmune disorders, as well as in combating senescence. Sources of therapeutic NK cells include peripheral blood, umbilical cord blood NK cells, stem cell-derived NK cells, NK cell lines, adaptive NK cells, cytokine-induced memory-like NK cells, and chimeric antigen receptor NK cells [202]. NK cells can be engineered to express CARs directing these cells against specific ligand-expressing cells. CAR-NK cells exhibited remarkable efficacy in treating multiple myeloma [203]. Compared to CAR-T cells, CAR-NK cells do not elicit CRS and persist for a shorter time in the body, reducing the risk of graft-versus-host disease [204]. As for their use against senescence, Bai et al. showed that adoptive transfer of NK cells significantly reduced aged CD3^+^ T cells in the peripheral blood [205]. NKG2DLs could be used as targets for anti-aging therapy with CAR-NK cells [165]. However, research on CAR-NK cells against aging is only in its infancy.

Unfortunately, under normal conditions, NK cells are rare in the brain parenchyma, but may be found in the choroid plexus and meninges [206]. They cross the BBB more easily if weakened by inflammatory conditions [207]. Nonetheless, CAR-NK cells can be engineered to express receptors that match inflammatory chemokines [208] or adhesion molecules that interact with the endothelium to promote their migration into the CNS [209]. Loading NK cells into vesicles that cross the BBB or magnetic nanoparticle guidance could also be employed to improve their CNS penetrance [210,211].

### 5.3. CAR-M Cell Therapy Against Senescent Cells

Macrophages produce inflammatory cytokines such as TNF-α, IL-1β, IL-6, or IL-12, with crucial roles in regulating immune responses. However, with aging, the efficiency and selectivity of macrophages tend to diminish. By using CARs, macrophages can be instructed to recognize specific antigens on malignant cells [152], a strategy currently evaluated in 2 clinical trials—NCT046602-929 and NCT05164666 [153]—or on senescent cells. Aside from their phagocytic abilities, CAR-M cells enhance antigen presentation and activate cytotoxic T cells [212]. Like CAR-NK cells, CAR-M cells are less toxic and have a short lifetime. Future research will evaluate their potential as an anti-aging strategy, but they seem to be a promising option.

### 5.4. CAR-iNKT Cell Therapy Against Senescent Cells

iNKT cells can be activated by binding a glycolipid antigen such as α-galactosyl ceramide to its HLA class I-like molecule CD1d [213]. However, to enhance their rapid targeting of senescent cells, “navigation” CARs can be introduced into iNKT cells, enabling them to regulate immune responses in extralymphatic tissues [214]. Although iNKT cells are a rare subset of lymphocytes, their robust in vitro proliferative capacity makes them a promising therapeutic tool [215]. Nonetheless, their potential anti-aging properties will need to be validated by future research.

**Figure 3:**
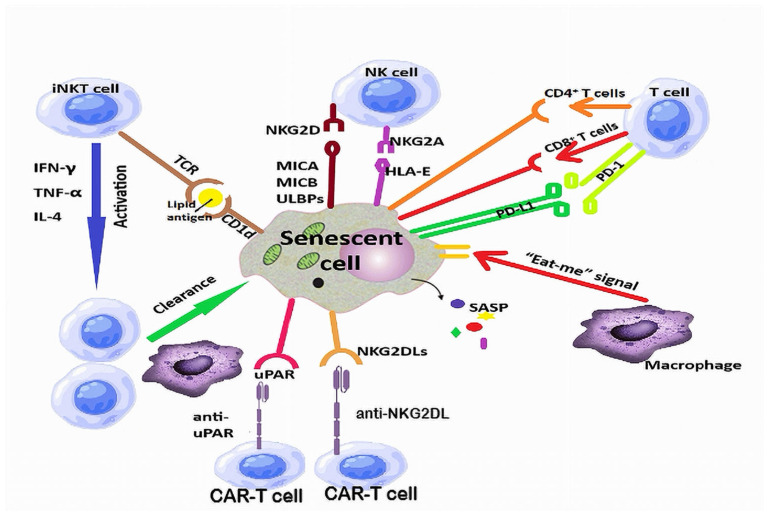
Immune cells and immune activation mechanisms involved in the elimination of senescent cells. Each immune cell subtype relies on the recognition and binding of specific antigens expressed by senescent cells. CAR-T cells have so far been generated harboring receptors for urokinase plasminogen activator (uPAR) and natural killer group 2 member D ligands (NKG2DLs).

The possibilities of CAR cell therapy against senescent cells are shown in Figure 3.

## 6. The Potential of Cell Therapy in Alzheimer’s Disease

Alzheimer’s disease (AD) is a progressive neurodegenerative disease leading to profound cognitive impairment caused by the deposition of extracellular amyloid β plaques (Aβ) and intracellular accumulation of neurofibrillary tangles, which ignite neuroinflammation [15,216,217]. Current treatments provide only symptomatic relief, and the scientific community has struggled to find disease-modifying treatments for AD.

The recent approval of three monoclonal antibodies against Aβ by the FDA and EMA was a breakthrough in the treatment of AD. However, they slow cognitive decline by only about 30% [218], with a high risk of adverse events due to amyloid-related imaging abnormalities (ARIA) [219]. Based on the imaging aspect, ARIA is subdivided into edema/effusion ARIA (ARIA-E), occurring in 0.1–0.8% of patients, and microhemorrhages and superficial siderosis (ARIA-H), with a prevalence of around 23% [219]. The pathophysiology of ARIA is believed to be related to the breakdown and mobilization of Aβ aggregates from the brain parenchyma into the vasculature, increasing the progression of cerebral amyloid angiopathy [220] and binding of anti-Aβ antibodies to Aβ in the vasculature, disrupting its integrity [221].

Given these limitations of passive immunization strategies in AD, cell therapies have emerged as attractive alternatives. CAR-T cells or CAR-Ms could selectively remove senescent and dysfunctional microglia and astrocytes, thereby resetting the inflammatory microenvironment and allowing for healthier immune surveillance. Moreover, engineered T cells could be designed to recognize tau-expressing neurons and selectively clear them, while macrophages could recognize and phagocytize Aβ plaques more efficiently than microglial cells. However, these treatments would work best if initiated early in the disease course and not in advanced stages. Currently, the scientific world struggles to find reliable, cost-effective, and non-invasive biomarkers that would allow an early diagnosis of AD and initiation of treatment [222].

The choice of the proper cell type is crucial. Research has shown that effector T cells react to the misfolded Aβ aggregates and clonally expand, promoting neuroinflammation and increasing AD neuropathology [111]. Moreover, adoptive transfer of Aβ-reactive effector T cells accelerates cognitive impairment and amyloid pathology in APP/PS1 mice [116]. Tregs maintain immunological tolerance during disease, and adoptive transfer of polyclonal Tregs has beneficial effects in several neurodegenerative diseases, including AD [223]. Unfortunately, polyclonal Tregs could cause global immune suppression and increase the prevalence of infectious diseases or malignancies. Immune dysregulation could be overcome using Tregs specific for certain pathological proteins such as Aβ. Engineering CAR-Tregs to release neurotrophic factors such as brain-derived neurotrophic factor or nerve growth factor when engaging amyloid or tau could promote neuronal survival and synaptic plasticity. As such, CAR cell therapy would qualify as a disease-modifying therapy exhibiting regenerative effects. In addition, other engineered cell types, such as macrophages or NK cells, are actively evaluated.

### 6.1. Aβ-Specific Tregs in Alzheimer’s Disease

None of the therapeutic approaches discussed below have escalated to clinical trials, but the results of in vitro and animal experiments are encouraging.

Saetzler and coworkers generated an Aβ-specific scFv that was cloned into a second-generation CAR construct with a CD8 hinge and an intracellular CD28 costimulatory domain and CD3ζ activation domain [224]. The resulting CAR-Treg cells showed a normal phenotype, normal proliferative capacity, were Aβ-specific, could be activated in vitro by both recombinant and membrane-bound Aβ isolated from murine brain, and were able to suppress activated T cells.

Park and coworkers isolated and expanded CD4^+^CD25^+^ Tregs after Aβ stimulation and transferred them into 5xFAD mice. The approach led to improved cognitive function and reduced Aβ and phosphorylated tau accumulation in the hippocampus of the treated animals, also modulating neuroinflammation as shown by the mRNA expression of proinflammatory markers such as TNF-α, IL-1β, IL-6, and nitric oxide synthase 2 [123].

Yeapuri and coworkers generated Aβ-specific Tregs by CRISPR/Cas9 knockout of endogenous TCR and incorporation of the transgenic TCR identified in Aβ-reactive effector T-cell clones. Following the adoptive transfer of the engineered cells, APP/PS1 mice showed improvements in cognitive functions and a reduced amyloid load. The treatment also improved glucose uptake, and the Aβ-specific Tregs easily infiltrated the brain, exhibiting homing properties. They also reduced reactive microglia, as shown by reduced glial fibrillary acidic protein (GFAP) expression [18].

Using already available antibodies targeting tau (E2814), Aβ (Lecanemab and Aducanumab), and truncated Aβ_3–42_ (Donanemab and Remternetug), Siebrand and coworkers showed that the CARs can detect and discriminate between fibrillar tau, Aβ_1–42_, and Aβ_3–42_ in cell systems, recognizing pathologic and physiologic conformers [225]. All the engineered cells produced IL-2 when stimulated by the corresponding antigen. Moreover, the research confirmed the cytotoxic effect of Aβ, the cells showing increased proliferation at low concentrations but significantly decreased viability when exposed to high concentrations of amyloid beta [225].

### 6.2. Chimeric Antigen Receptor Macrophages in Alzheimer’s Disease

Because in AD, as in other neurological diseases, microglia undergo phenotypical changes that are detrimental to disease progression, researchers have tried to replace dysfunctional microglia with microglial transplants or peripheral monocytes [226]. Evidence suggests that peripheral monocytes/macrophages can degrade and phagocytose Aβ plaques more efficiently than microglia [227]. Moreover, recent reports even suggest that microglia may promote the spread of Aβ plaques [228]. Allogenic CAR-M therapy could overcome dysfunctional cell-mediated clearance of amyloid plaques in AD.

After demonstrating the recruitment of peripheral monocytes/macrophages by amyloid plaques in APP/PS1 transgenic mice [229], researchers tried to increase the cells’ ability to resorb Aβ by inserting a CAR using Aducanumab as scFv. The engineered macrophages performed well in vitro and in brain slices, but had limited survival in vivo. As such, a second-generation “reinforced CAR-M” that produces M-CSF to enhance its own survival was generated and found to efficiently resorb plaques after local delivery in the hippocampi of aged APP/PS1 mice [17]. The use of CAR-Ms may also have several advantages over anti-Aβ monoclonal antibodies, such as (a) the possibility to be engineered to express enzymes that help degrade amyloid, (b) their ability to degrade plaques that does not rely on antibodies or resident microglia for Fc mediated uptake, and (c) the ability to degrade rather than redistribute amyloid, which is likely to reduce the incidence of ARIA [17].

## 7. Limitations, Challenges, and Future Perspectives

CAR cell therapy has emerged as a rapidly developing and promising therapeutic strategy for malignancies, allowing for personalized immunotherapy. Following their success in hematological cancers, their potential is beginning to be evaluated in senescence and age-related neurodegenerative diseases such as AD. However, several limitations need to be overcome to improve clinical outcomes.

First, the cell type should be carefully chosen. For CAR-T cells, existing clinical data show that CD4^+^ T cells have better persistence compared to CD8^+^ T cells, and autologous cells are detectable for longer periods in vivo than allogenic T cells [230,231]. Culture conditions can also influence the proliferation and differentiation of CAR-T cells. For example, adding cytokines such as IL-2, IL-7, IL-15, and IL-21 boosts T-cell proliferation and leads to the formation of more effector T cells [232]. However, the risk of CRS and cognitive impairment following CAR-T therapy could seriously limit its application in neurological diseases. CAR-NKs could be a more favorable alternative, carrying a lower likelihood of CRS due to the different secretomes of activated NK and T cells. Furthermore, NK cells can be derived from cell lines or induced pluripotent stem cells (iPSCs) with less stringent requirements for autologous immune cells and less risk of graft-versus-host disease in the allogenic setting [233]. This could also significantly lower the cost of the therapy, favoring “off-the-shelf” production [233].

Second, a major hurdle in CAR cell-related technologies is the scarce knowledge of selective markers able to identify senescent cells. Currently, research has focused on 2 antigens, uPAR and NKG2DL, and the assessment of the treatment effect relied on demonstrating the reduction of senescent cells as indicated by single markers such as p16 and SA-β-gal, which may not accurately reflect the cell populations targeted by the senolytic CAR-T cells [233]. In addition, the antigens targeted by CARs are involved in intracellular signaling and regulation of the immune response. As such, future research should investigate the long-term effects of such therapies on health span and lifespan, a particularly relevant issue considering the persistence of CAR-T cells in vivo.

Third, ensuring that CAR cells reach the brain in sufficient numbers and persist long enough to have sustained effects is another major challenge [234]. Studies focusing on glioblastoma or brain metastases concluded that local delivery outperforms IV infusion of engineered immune cells [235,236]. While aggressive delivery methods could be accepted in cancer patients, such invasive means of delivery would be unacceptable in frail elderly patients, which is why several strategies are under development. Up-tuning integrins/selectin ligands such as VLA-4 (very late antigen 4), PSGL-1 (P-selectin glycoprotein ligand-1), or α4β1 integrin promotes firm adhesion of the construct to the activated brain endothelium and transendothelial migration [199]. Adding transferrin receptor- or insulin receptor-directed single-chain binders to CAR constructs leverages receptor-mediated transcytosis [237]. For enhanced persistence of CAR-T cells, using naïve, less differentiated T cells [238] or using specific culture conditions (such as stimulating the dividing cells with IL-7 and IL-15 instead of IL-2) could improve their persistence [239]. The overexpression of transcriptional regulators that oppose exhaustion (for example, c-Jun) [240] or armoring the CAR-T cell construct with supportive cytokines [241] also enhances persistence. Alternatively, metabolic reprogramming of the engineered cells to enhance mitochondrial biogenesis targeting PGC-1α leads to similar results [242].

Artificial intelligence (AI) can be an invaluable tool in optimizing CAR cell-based therapy against aging and AD. By sifting through large-scale proteomic and genomic data, AI could identify novel antigens uniquely expressed on senescent cells. Further, it could analyze large databases of antigen structures and T-cell interactions to predict the most effective CAR constructs, as well as predict treatment success and potential side effects in both senescence and AD [243].

CRISPR/Cas9 is another innovative technology that could revolutionize the treatment of many diseases. Specifically for AD, it is used in constructing AD models, screening for pathogenic genes that increase the risk for AD, or used as gene therapy targeting genetic mutations, key enzymes involved in amyloid precursor protein processing, or proinflammatory molecules such as CD33 or cysteinyl leukotrienes (Cys-LTs) [244].

However, the limitations of CAR-T cell therapy, such as CAR-T cell exhaustion and CRS, have prompted the scientific community to search for novel strategies to overcome these challenges. Exosomes, composed of a lipid bilayer membrane and released by various cells, exchange information with recipient cells via cytoplasmic division, membrane fusion, and receptor-mediated transport [245,246]. Exosomes released from CD47-expressing cells bind to the receptor signaling regulator protein α (SIRPα) and prolong the activity of macrophages in vivo by facilitating the evasion of immune clearance [247]. Exosomes released by NK cells contain granzyme B and perforin, which boost the body’s immunity [248]. Compared to CAR-T cells, exosomes have superior biocompatibility and loadability. Moreover, exosomes derived from multi-targeted CAR-T cells likely have multiple surface targets and are accordingly more effective but safer for patients [143].

## 8. Conclusions

Although threatened by the increasing number of elderly persons living worldwide and the rapid rise in the prevalence of dementia, the scientific community and healthcare systems do not yet have efficient means to combat senescence or dementia and rely mainly on preventive measures such as control of vascular risk factors [249] and lifestyle changes. It is now widely accepted that immunosenescence contributes to a certain extent to age-related frailty and is involved in the pathophysiology of AD. A series of lifestyle factors recommended as preventive means also influence the immune system. Regular exercise and caloric restriction reduce immune senescence in elderly mice by reversing age-induced alterations in CD4^+^ T naïve and effector subsets [250]. Yoga exercises help maintain a homeostatic balance between Th17 and Treg cells [251]. Aside from its antioxidant properties [252], resveratrol increases the number of CD4^+^ T cells in the thymus and delays atrophy of this organ in mice treated with D-galactose [253]. The senolytic combination dasatinib + quercetin [176] induces the differentiation of CD4^+^ T cells towards a more youthful phenotype [254].

In addition to the aforementioned approaches, the potential of CAR cell therapy is real, and this strategy is a leap toward precision medicine for neurodegenerative diseases. Genetic analyses to identify persons at risk for developing AD and early identification of AD by using circulating biomarkers [222] would allow for initiation of therapy aiming to salvage neuronal cells from degeneration in more incipient stages of the disease. Eliminating senescent cells once an age-related condition is identified could reduce the progression of senescence and reduce generalized low-grade inflammation in the entire body [5]. However, research is still in the preclinical stage and must address a series of critical steps, such as improving delivery mechanisms and ensuring long-term safety, before transitioning to controlled clinical trials.

## Figures and Tables

**Table 1:** Genetic loci linked to the inflammatory intracellular signaling pathways and shown by GWASs to influence the carrier’s susceptibility to AD.

Gene	Gene Product	Role in Neuroinflammation	Polymorphisms	Effect	References
*TREM2*	TREM2 (triggering receptor expressed on myeloid cells 2)	Microglial phagocytosis	*R47H* variant	Increases neuritic dystrophy caused by Aβ deposition and reduces microglial function in Caucasians	[78,79]
			*R63H* variant	Increases the risk for AD in individuals of European ancestry	[80]
			*H157Y* variant	Increases the shedding of the soluble form of the receptor and decreases cell surface expression of the receptor; increases the risk for AD mainly in Chinese populations	[80]
*CD33*	A transmembrane receptor on the myeloid lineage cells that belongs to the SIGLECs (sialic acid-binding Ig-superfamily of lectins)	Inhibits microglial amyloid clearance	SNP rs3865444	Protective effect by promoting Aβ_42_ clearance by microglial cells	[81]
*CR1*	Complement component (3b/4b) receptor 1	Complements activation, synapse pruning	SNP rs6656401	Increases the susceptibility for late-onset AD	[82]
*CLU*	Clusterin, an astrocytic protein with anti-amyloidogenic functions	Complements regulation, immune recruitment	rs11136000	Increases the risk for AD mainly in Caucasians	[83]
*PILRA*	PILRA (paired immunoglobulin-like type 2 receptor alpha), a microglial surface inhibitory receptor	Microglial inhibitory signaling	G78R (rs1859788)	Protects carriers from the risk of AD	[84]
*EPHA1*	EPHA1 (ephrin type-A receptor 1)	Recruitment of immune cells	P460L variant	Increases the risk for late-onset AD	[85]
*ABI3*	ABI3 (ABI interactor family member 3)	Microglial cytoskeletal regulator; impacts motility and microglial response to Aβ plaques	SNPs rs5978930 and rs16947151	Increase the risk of AD, associated with faster cognitive decline	[86]
*HLA-DRB1*	Beta chain of the human leukocyte antigen class II molecule	Involved in antigen presentation to CD4^+^ T cells and microglial activation	SNP rs9271192	Associated with late-onset AD	[87]
*MS4A6A*	Membrane spanning 4-domains A6A	Involved in the immune clearance of Aβ	rs610932	Associated with late-onset AD	[88]
*ABCA7*	ATP-binding cassette sub-family A member 7	Regulates the communication between microglia and astrocytes through the NLRP3 inflammasome and the release of proinflammatory cytokines	rs3764650	Increases the risk for AD	[89]

**Table 2:** Surface markers of senescent cells (adapted from ref. [165]).

Surface Marker	Cell Type	Aging Model and Detection Pathway	Reference
B2M	Bladder cancer cells	Senescence—induced by doxorubicin	[186]
NOTCH1	Fibroblasts	Senescence was induced by mitogen-activated extracellular signal-regulated kinase (MEK) and DNA damage	[187]
PD-L1	fibroblasts	Senescence was induced by DNA damage and Nutlin-3	[64]
uPAR	Liver fibrosis cells and lung adenocarcinoma cells	Senescence induced by replication induction, MEK and CDK4/6 inhibitors, liver fibrosis induced by CCl_4_	[188]
GPNMB	Vascular endothelial cells	Senescence was induced by feeding a high-fat diet	[182]
CD38	Alveolar epithelial cells	Lung fibrosis was induced in mice by intratracheal bleomycin	[189]
CD26 (DPP4)	Human cartilage	Chondrocytes were acquired from individuals undergoing total knee replacement; research evaluated co-expression of DPP4 with established senescence markers by flow cytometry, and expression levels of anabolic and catabolic genes, senescence related genes and SASP phenotypes in DPP4^+^ and DPP4^−^ cells	[190]
CD47	Fibroblasts	Premature senescence was induced with hydrogen peroxide	[191]
DcR2	Renal tubular epithelial cells	Renal fibrosis was induced with streptozotocin in mice with diabetic nephropathy	[192]
Oxidized vimentin	Human chondrocytes	Doxorubicin-based in vitro model of stress-induced premature senescence	[193]
CD44	Vascular endothelial cells	Replicative senescence induced by serial passages	[194]
MICA, MICB, ULBP2 (NKG2D ligands)	Lung fibroblasts	Senescence was induced by DNA damage, replication deletion, and triggering oncogenes	[195]

Abbreviations: B2M—beta 2 microglobulin; NOTCH1—neurogenic locus notch homolog protein 1; PD-L1—programmed cell death protein 1-ligand 1; uPAR—urokinase plasminogen activator receptor; GPNMB—glycoprotein non-metastatic melanoma protein B; DPP4—dipeptidyl peptidase 4; MICA—MHC class I polypeptide-related sequence A; MICB—MHC class I polypeptide-related sequence B; ULBP2—UL16 binding protein 2.

## Data Availability

No new data were generated during the writing of this review.

## Abbreviations

The following abbreviations are used in this manuscript:

Aβ	amyloid beta
ABCA	ATP-binding cassette subfamily A
AD	Alzheimer’s disease
AI	artificial intelligence
Akt	protein kinase B
ALL	acute lymphoblastic leukemia
ARIA	amyloid-related imaging abnormalities
B-ALL	B-cell acute lymphoblastic leukemia
BBB	blood–brain barrier
BBIR	biotin-binding immune receptor
BCR	B cell receptor
BTK	Bruton’s kinase
CAR	chimeric antigen receptor
CAR-Ms	chimeric antigen receptor macrophages
CAR-NKs	chimeric antigen receptor natural killer cells
CAR-T	CAR-T cells
CAR-Tregs	chimeric antigen receptor regulatory T cells
Cas9	caspase-9
CD	cluster of differentiation
CLL	chronic lymphocytic leukemia
CNS	central nervous system
CR1	complement component (3b/4b) receptor 1
CRISPR	clustered regularly interspaced short palindromic repeats
CRS	cytokine release syndrome
CSF	cerebrospinal fluid
DAP12	DNAX activation protein of 12 kDa
DLBCL	diffuse large B-cell lymphoma
DNA	deoxyribonucleic acid
EM	effector memory (T cells)
EMA	European Medicines Agency
EphA1	ephrin type-A receptor 1
FDA	Food and Drug Administration
FL	follicular lymphoma
FoxP3	forkhead box P3
GM-CSF	granulocyte-macrophage colony-stimulating factor
GWAS	genome-wide association study
HAMA	human anti-mouse IgG antibody
HLA	human leukocyte antigen
ICANS	immune cell-associated neurological syndrome
IFN	interferon
IL	interleukin
iNKT	invariant natural killer T cells
iPSCs	induced pluripotent stem cells
KLRG	killer cell lectin-like receptor subfamily g
LBCL	large B-cell lymphoma
mAb	monoclonal antibody
MAPK	mitogen activated protein kinase
MCL	mantle cell lymphoma
MCP-1	monocyte chemoattractant protein-1
M-CSF	macrophage colony stimulating factor
MEK	mitogen-activated protein kinase
MHC	major histocompatibility complex
MICA	MHC class I chain-related protein A
MICB	MHC class I chain-related protein B
mRNA	messenger ribonucleic acid
mTOR	mammalian target of rapamycin
NFAT	nuclear factor of the activated T cell
NF-κB	nuclear factor- κB
NK	natural killer T cells
NKG2A	member A of the G2 receptor family of natural killer cells
NKG2DL	natural killer group 2 member D ligand
NRF1	nuclear respiratory factor 1
PD-1	programmed death-1
PD-L1	corresponding ligand for the PD-1 receptor
PGC-1α	peroxisome proliferator-activated receptor gamma coactivator 1-alpha
PI3K	phosphatidylinositol-3-kinase
PILRA	paired immunoglobulin-like type 2 receptor alpha
PMBCL	primary mediastinal large B-cell lymphoma
RUNX3	Runt-related transcription factor 2
SASP	senescence-associated secretory phenotype
scFv	single-chain variable fragment
SIGLECs	sialic acid-binding Ig-superfamily of lectins
SIRPα	signal-regulated protein α
SLL	small lymphocytic lymphoma
sMAC	sestrin-activated MAPKinase activation complex
SNP	single-nucleotide polymorphism
S1P	sphingosine 1-phosphate
S1PR1	sphingosine 1-phosphate receptor-1
STAT	signal transducer and activator of transcription
TCF1	T-cell factor 1
TCR	T-cell receptor
TEMRA	terminally effector memory re-expressing CD45RA
TFAM	mitochondrial transcription factor protein A
TGF-β	transforming growth factor-β
Th	T helper cell
ThPOK	T-helper-inducing POZ/Krueppel-like factor
TN	naïve T cells
TNF-α	tumor necrosis factor alpha
Treg	regulatory T cell
TREM2	triggering receptor expressed on myeloid cells 2
TRIB2	Tribbles homolog 2
TRUCK	T redirected universal cytokine killer cell
UCAR	universal CAR-T cell
ULBP2	UL16 binding protein 2
uPAR	urokinase plasminogen activator receptor
USA	United States of America
WHO	World Health Organization
